# Does urban density always boost smart productivity? Evidence of an inverted U-shaped relationship in Chinese cities

**DOI:** 10.1371/journal.pone.0326606

**Published:** 2025-06-24

**Authors:** Tao Chen, Yike Zhang, Yaoning Yang, Binbin Wu, Genyu Xu

**Affiliations:** Department of Urban and Rural Planning, School of Architecture and Planning, Yunnan University, Yunnan, China; Thammasat University, THAILAND

## Abstract

Does urban density always boost smart productivity? Based on panel data from 28 major Chinese cities (2011–2021), this study reveals an inverted U-shaped relationship between urban density and smart productivity. Using entropy weight method, we construct comprehensive indices to measure both urban density and smart productivity levels. Our findings demonstrate that urban density positively influences smart productivity up to a threshold of 0.497, beyond which the relationship becomes negative. The results from fixed effects modeling show that a 1% increase in urban density is associated with a 0.114% increase in smart productivity before reaching the threshold. Through mediation analysis, we find that urbanization level serves as a significant mediator, accounting for 49.1% of the total effect. Furthermore, heterogeneity analysis reveals distinct regional patterns: urban density exhibits stronger positive effects in western regions (coefficient = 0.181) compared to central regions (coefficient = 0.156), while showing negative impacts in eastern regions. These findings suggest that optimal urban density levels vary across regions, and cities should adopt differentiated development strategies accordingly. Our study contributes to the literature by quantifying the non-linear relationship between urban density and smart productivity, while providing empirical evidence for urban planning policies.

## 1. Introduction

### 1.1 Research background

Within the traditional framework of Marxist political economy, productivity is conceptualized as humanity’s capacity to utilize and transform nature, representing the synthesis of social and natural forces through organized labor [1]. Smart Productivity (SP), alternatively termed as New Quality Productive Forces (NQPF) by the Chinese government, fundamentally belongs to the domain of productivity development. However, it distinguishes itself from traditional productivity through its distinctive characteristics of innovation, digitalization, environmental sustainability, and enhanced labor quality [1].

Li and Cui [2] further elaborated that Smart Productivity represents an advanced form of productive capability, characterized by qualitative improvements in both overall productivity and its constituent elements. These elements encompass new quality labor force, new quality labor objects, and new quality labor materials [3].

A significant distinction exists between traditional productivity and Smart Productivity. While the former primarily emphasizes the utilization of labor and material resources through conventional production methods and technical means, Smart Productivity prioritizes scientific and technological innovation alongside digital transformation. This emphasis facilitates the development of highly efficient, coordinated, and sustainable production systems [4].

Based on the research mentioned above, it is evident that the main concept of new-type productivity emphasizes the core role of scientific and technological innovation, particularly in areas such as information technology, digital transformation, and intelligent manufacturing. These characteristics indicate that productivity no longer solely relies on traditional labor and material resources, but is instead driven by the application of innovative technologies, leading to significant leaps in productivity. This mainly involves industries such as artificial intelligence, big data, smart manufacturing, digital economy, cloud computing, and blockchain. Another core feature of new-type productivity is digitization, which is primarily manifested in the digitalization of production processes, the intelligence of management, and the high degree of informatization of supply chains. The widespread application of digital technologies has enhanced production efficiency, optimized resource allocation, and driven fundamental transformations in production models. Additionally, new-type productivity not only focuses on improving production efficiency but also emphasizes the environmental sustainability of the production process. By promoting the development of green technologies, such as clean energy, low-carbon production methods, and environmental protection technologies, it ensures the harmonious coexistence of the production process and the natural environment. Therefore, it can be summarized that the difference between new-type and traditional productivity lies in the following: traditional productivity emphasizes the direct use of labor and material resources to enhance productivity, whereas new-type productivity places scientific and technological innovation and digital transformation at its core. The core of traditional productivity is the combination of material and labor, while new-type productivity emphasizes knowledge, technology, and innovation-driven production models. Therefore, this paper defines smart productivity (or new-quality productivity) as a modern advanced form of productivity driven by technological innovation, characterized by revolutionary technological breakthroughs, innovative allocation of production factors, deep industrial transformation, and a commitment to sustainable development. Its essence lies in promoting high-quality development through the improvement of total factor productivity (TFP).

In the contemporary era, China has entered a new phase of development. Along with its rapid economic growth and accelerated industrialization process, labor costs have risen substantially. Consequently, numerous labor-intensive industries have relocated to developing countries in Southeast Asia [5]. To ensure sustainable domestic economic development, there is an imperative need to transform the previous development model, which heavily relied on labor-intensive industries, and establish new developmental directions and industrial transformation strategies.

The importance of new-type productivity is also reflected in the measures taken by the Chinese government. In response to the aforementioned challenges, the Chinese government has made numerous efforts, such as the National Development and Reform Commission (NDRC) of China promulgated a pivotal policy document in July 2023, entitled “Accelerating the Development of New-Quality Productive Forces”, which delineated specific strategic directives encompassing the enhancement of scientific and technological innovation, industrial advancement, green economy development, and institutional reform optimization [6]. Subsequently, in 2024, the NDRC issued a complementary document, “Cultivating and Developing New-Quality Productive Forces to Stimulate New Momentum for High-Quality Development,” which provided a comprehensive theoretical framework elucidating the scientific underpinnings of new-quality productive forces in facilitating economic development [7].

The development of Smart Productivity needs to rely on a certain scale of material production space, and extend the connotation of “new” on the basis of the traditional productivity development model. Smart Productivity in the context of scientific and technological innovation to achieve the enrichment and development of the category of workers, based on the digital context to achieve the connotation of the object of labor extension [1]. It is also based on the human brain innovation to further strengthen the importance of the labor tools to provide new tools for the production of new economic space support [8]. With the scientific and technological innovation, digital upgrading, human brain innovation to produce new economic space depends on the material space represented by the social and physical space. Cities are the main material space dependent on economic development, have the main position in economic development on the main concentration of production capacity. Therefore, there is an emerged need to explore the impact of urban spatial patterns on the development of new productive capacities.

The evolution of Smart Productivity necessitates a substantial material production space while expanding the “new” dimensions beyond traditional productivity development paradigms. Within the context of scientific and technological innovation, Smart Productivity facilitates the enrichment and development of the workforce category, while simultaneously extending the connotation of labor objects through digital transformation [1]. Furthermore, it emphasizes the significance of labor tools through cognitive innovation, thereby providing novel instruments for creating new economic spaces [5].

The emergence of new economic spaces, driven by scientific and technological innovation, digital transformation, and cognitive advancement, is fundamentally dependent on material spaces manifested in both social and physical dimensions. Cities, serving as the primary material spaces for economic development, occupy a paramount position in concentrating productive capabilities. Consequently, there is an emerging imperative to investigate the correlation between urban spatial configurations and the development of new productive capacities.

### 1.2 Literature review

#### 1.2.1 Urban density research.

To address the aforementioned challenges, scholars have conducted extensive research on compact cities and urban density. Initial academic investigations primarily concentrated on conceptualizing compact cities and developing methodologies for measuring urban density [9–14]. The concept of compact city, first introduced in 1973, garnered significant academic attention and spawned numerous studies across various countries and regions [9]. Jenks et al. [11] conceptualize the compact city paradigm as characterized by high urban density in 1996, mixed land use patterns, diverse functionality, efficient transportation infrastructure, enhanced pedestrian accessibility, and socio-economic heterogeneity.

Subsequent research has further refined these analytical frameworks. Fang and Qi [12] synthesized a comprehensive index system for measuring urban compactness, establishing a more scientifically rigorous methodology for evaluating urban density in 2005. Stevenson et al. [13] advanced this field by simulating compact city scenarios based on increased land use density and diversity in 2016, while considering reduced distances to public transportation infrastructure. Jin et al. [14] developed an urban density evaluation framework, implementing it in a comparative analysis of Guangzhou’s Zhujiang New City and Hong Kong’s Central district in 2018. Recent studies have demonstrated that compact city development has contributed significantly to enhanced urban efficiency and economic development [15–19], establishing itself as a crucial urban development paradigm aligned with contemporary objectives of quality and efficiency improvement.

This extensive body of international research on compact cities, approaching the subject from diverse perspectives, has consistently validated the efficiency-enhancing effects of urban compactness on development. However, a critical question remains: whether compact city development can effectively address the pressing need for “Smart Productivity” development. This theoretical and practical conundrum demands urgent attention. Resolving this query is vital not only for elucidating the specific mechanisms through which urban density influences Smart Productivity but also for providing strategic insights into urban development pathways that facilitate economic model transformation and capitalize on emerging opportunities in the new era.

#### 1.2.2 Smart productivity studies.

While research examining the relationship between Urban Density and Smart Productivity remains relatively limited, scholarly investigations into Smart Productivity itself have yielded substantial insights. The existing literature on Smart Productivity primarily encompasses three fundamental dimensions: theoretical conceptualization, principal characteristics, and interrelationships with various factors [20–24].

Existing scholarship [20,21] proposes that smart productivity evolution constitutes a process of attaining critical technological breakthroughs and disruptive innovations while maintaining continuity with conventional productivity frameworks [20]. Its fundamental transformation manifests through optimized syntheses of labor forces, production materials, and operational objects [21]. Complementing this theoretical foundation, research on NQPF characteristics [22,23] delineates contemporary manifestations of smart productivity in emerging developmental phases, particularly highlighting its digital transformation, intelligent automation, network integration, and data-centric operational paradigms [22]. Furthermore, this progression aligns with strategic economic liberalization initiatives that systematically enhance productivity advancement pathways [23].

Empirical methodologies for assessing Smart Productivity development have gained prominence, with methodological advances including the implementation of comprehensive indicator systems and systematic assessments of regional development disparities [24]. Contemporary Chinese research on Smart Productivity predominantly focuses on its applications in energy systems, industrial development, and sustainability [25–27]. These studies aim to elucidate the influence mechanisms of Smart Productivity correlates, offering valuable comparative insights to domestic research frameworks.

#### 1.2.3 Knowledge gaps of two fields.

Based on the comprehensive review of existing literature, while studies on urban density have highlighted the positive effects of compact cities on enhancing urban efficiency and economic development, there has been insufficient discussion regarding potential negative impacts, such as environmental pressures, traffic congestion, and social inequalities that may arise from excessive density. Furthermore, although existing research mentions global studies on compact cities, the analysis of regional variations remains limited. For instance, the impacts of urban density on economic, social, and environmental aspects may vary significantly across different countries and regions, warranting further investigation.

Research on smart productivity has primarily focused on three aspects: theoretical connotations, main characteristics, and logical relationships with other factors. These studies have provided multidimensional definitions of the core implications of smart productivity and developed methodological support for its quantitative evaluation through the construction of indicator systems. However, existing literature on smart productivity predominantly remains theoretical, lacking substantial integration with empirical research. The specific correlations between smart productivity and factors such as urban economic structure optimization and production efficiency require more in-depth exploration.

Consequently, it is evident that research on the relationship between urban density and smart productivity remains relatively scarce, and relevant theories need urgent improvement. Although the development of compact cities is considered conducive to enhancing urban efficiency, and smart productivity emphasizes qualitative leaps through technology and innovation, the specific connections and mechanisms between these two aspects have yet to be clearly established. The main research directions in two fields are summarized in Table 1.

**Table 1:** The main research directions in two fields.

	Research hotspots	Research frontier
Smart Productivity	High-quality development, Scientific and technological innovation or Tech innovation, Digital economy	Digital technology, Modern industrial system, Big data
Urban Density	Urban form, Urban expansion, Carbon emissions, Spatial form, Influencing factors, Urban space	Carbon emissions, Regional integration, Influencing factors, Urban sprawl

### 1.3 Research contributions

Despite extensive literature on urban economics, there remains a significant lacuna in our understanding of the causal mechanisms through which Urban Density influences Smart Productivity. This study advances the existing scholarship by employing a mixed-methods approach to investigate this relationship through several interconnected analytical dimensions.

The methodological framework encompasses four primary components: First, we develop a comprehensive indicator system to quantify both Urban Density and Smart Productivity metrics, subsequently incorporating these variables into an econometric model. Our empirical analysis utilizes panel data from Chinese municipalities and provincial capitals spanning 2011–2021, enabling a robust examination of the dynamic relationship between Urban Density and Smart Productivity. Second, drawing from theoretical foundations in urban economics, we employ mediation analysis to examine the intermediary role of urbanization rates in the relationship between Urban Density and Smart Productivity, thereby elucidating the underlying transmission mechanisms through which density influences productive capacity.

Third, having established the positive effect of Urban Density on Smart Productivity, we conduct a heterogeneity analysis by stratifying the sample cities into three geographical regions (eastern, central, and western China), allowing us to examine spatial variations in the density-productivity relationship. This geographic decomposition provides crucial insights into the contextual factors that moderate the effectiveness of urban density in promoting smart productivity.

Finally, based on our empirical findings, we advance policy recommendations for enhancing productivity within the framework of compact city development, considering the varying regional contexts and institutional environments. These recommendations are grounded in our empirical evidence and theoretical understanding of the urban density-productivity nexus.

## 2. Theoretical hypotheses

### 2.1 Direct effects of urban density

Marx’s foundational observation that “The productivity of labor is constantly developing with the continuous progress of science and technology” [25] provides the theoretical basis for understanding Smart Productivity. Within the framework of Marxist political economy, Smart Productivity represents an advanced form of productive forces, emerging through the continuous enhancement of productivity components [22]. This conceptualization requires the synchronized development of three essential elements: high-quality labor power, advanced means of production, and innovation-driven instruments of production, with each component undergoing qualitative improvements in both substance and form.

In the context of urban high-quality development, Smart Productivity assumes critical importance. Cities, as concentrated centers of capital resources and technological innovation, provide the institutional environment where market mechanisms operate effectively. These urban environments offer both optimal conditions and extensive opportunities for the cultivation, development, and practical application of Smart Productivity.

Based on an analysis of compact city characteristics and Smart Productivity conceptualization, the influence of compact cities on Smart Productivity primarily manifests through labor force concentration, efficient allocation and centralized utilization of labor objects, and enhanced urban innovation capacity. The four key dimensions – economy, population, land use, and transportation – affect labor subjects, objects, and tools, thereby inducing changes in Smart Productivity. The mechanism through which Urban Density impacts Smart Productivity is reflected in the following aspects:

Firstly, compact cities facilitate concentrated economic activities, generating economic agglomeration that promotes rapid convergence of enterprises and population factors toward the central city [26]. This concentration reduces information asymmetry within the urban environment, facilitating expedited factor matching, enhancing production efficiency, and consequently promoting Smart Productivity. The agglomeration of extensive economic activities enables infrastructure sharing and construction to achieve economies of scale, improving urban resource allocation efficiency [27]. The economic agglomeration-induced concentration of enterprise activities leads to the accumulation of high-skilled labor.

In comparison to sprawling cities, compact cities exhibit more diverse land use patterns and higher land use efficiency, contributing to increased average land output value and improved economic efficiency. These cities support varied production activities, accelerate capital circulation, diversify and expand labor objects’ forms and scope, and generate enhanced employment opportunities. These characteristics create more favorable conditions for Smart Productivity development.

Secondly, compact cities facilitate the concentration of talent, capital, and resources in urban centers, generating scale effects and enhancing urban economic vitality [28]. The heightened economic vitality attracts high-quality human capital, with research demonstrating that a city’s human capital stock significantly influences its attractiveness to well-educated individuals, recent university graduates, and potential migrants seeking higher economic opportunities [29], thereby creating a self-reinforcing cycle. The agglomeration of talent and capital generates knowledge spillover effects, which promote technological innovation, enhance urban productivity, and facilitate the transition toward high-technology industries, ultimately elevating the city’s Smart Productivity level.

Thirdly, compact cities exhibit concentrated traffic patterns, characterized by higher road network density and enhanced capacity for regional width, neighborhood density, and lane count [30]. Transportation system optimization enables high-density road networks to interconnect diverse urban functional zones, reducing commuting costs and enhancing residents’ daily convenience. The qualitative dimension of productive forces incorporates environmental considerations [31], establishing ecological and sustainability requirements for urban development. The compact integration of urban spatial structures and continuous transportation system optimization significantly reduces urban traffic carbon emissions, supporting urban green development [32].

However, The above inference does not prove that urban density will always have a positive impact on cities. Florida, R. (2017) argues that overly dense urbanization patterns can have negative effects on production efficiency, social inequality, and the environment [33]. The author suggests that while concentrated economic activities in cities can contribute to improved economic efficiency, excessive urban compactness may lead to pressures on transportation, housing, public services, and the environment, which in turn could affect urban productivity.

When urban compactness exceeds certain thresholds, it can negatively impact Smart Productivity, manifesting in “Urban Disease” [34]. This phenomenon weakens industrial scale effects, potentially reducing Smart Productivity. Urban diseases may precipitate multiple challenges: decreased external investment, diminished industrial diversity, reduced talent attraction, weakened labor target diversity, decreased capital mobility, high-quality labor shortages, and insufficient technological upgrading momentum, in 2020, Liu, Y., & Li, B. further proposed that excessively dense cities often face serious issues such as air pollution, traffic congestion, and resource waste, which in turn affect overall production efficiency and environmental quality [35]. This suggests that excessively high urban density is not conducive to sustainable development and may hinder the further development of new-type urban productivity. Furthermore, overcrowding leads to enterprise congestion and intensified competition, compelling firms to prioritize immediate competitive challenges over technological innovation investments. Excessive land and population density can severely impact public resource allocation, elevating residential living costs and potentially triggering high-quality labor outflow.

In summation, compact cities demonstrate characteristics of economic activity, talent, capital, and resource agglomeration. While compact spatial configuration and road networks enhance urban infrastructure, energy utilization, and land use efficiency—promoting industrial structure optimization and technological innovation, thereby advancing Smart Productivity—excessive density within certain parameters can induce urban diseases, affecting production efficiency, reducing capital investment, diminishing high-quality labor attraction, and potentially decreasing urban Smart Productivity.

**Fig 1:**
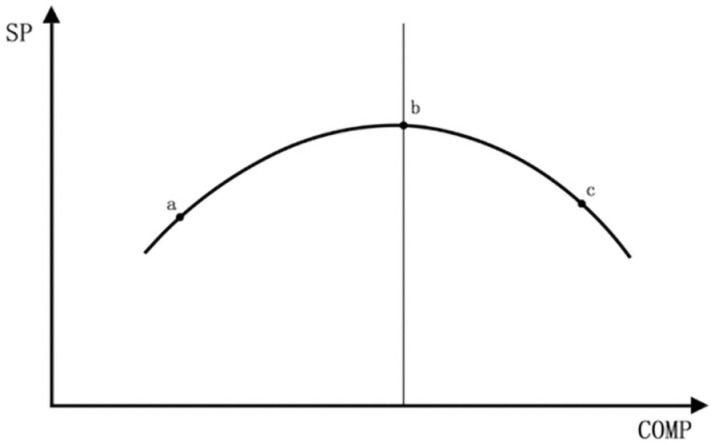
The inverted “U”-shaped curve illustration.

Consequently, this study proposes:

Hypothesis 1. The relationship between Urban Density and urban Smart Productivity exhibits an inverted “U” pattern (Fig 1).

As illustrated in Fig 1, the relationship between Urban Density and Smart Productivity exhibits an inverted U-shape. Initially, as Urban Density increases, economic activities and industrial distributions become more concentrated. This concentration, coupled with improvements in urban infrastructure, energy utilization, and land use efficiency, leads to an enhancement in Smart Productivity. However, upon reaching a certain threshold—denoted as point b in the Fig 1—the carrying capacity of the urban environment, population size, and scale of industrial agglomeration approach saturation, resulting in Smart Productivity attaining its peak. Beyond this point, further increases in Urban Density lead to a decline in Smart Productivity. Excessive Urban Density can overburden the city’s environmental carrying capacity, leading to congestion effects that deter investment, prompt business withdrawals, and cause labor force attrition, thereby diminishing Smart Productivity.

### 2.2 Analysis of mediation effect

#### 2.2.1 Level of urbanization.

Urban density catalyzes the agglomeration of economic activities through multiple mechanisms. This economic agglomeration generates scale effects, which further stimulate enterprise concentration within cities [16]. The increased enterprise density subsequently creates additional employment opportunities, attracting skilled labor and contributing to elevated urbanization levels. Moreover, compact urban development facilitates efficient transportation systems. Cities with high density prioritize public transit infrastructure, resulting in reduced commuting times and enhanced transportation efficiency—factors that incentivize rural-to-urban migration for employment and settlement. Furthermore, increased urban density enhances economic vitality [16]. This manifests through improved economic efficiency, diversified land use patterns, enriched commercial activities, and enhanced accessibility to resources, collectively attracting rural population influx.

**Fig 2:**
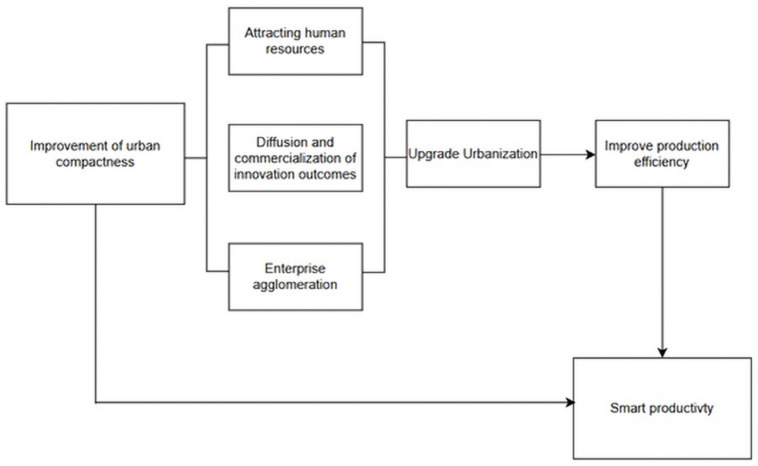
Mediation mechanism.

The fundamental nature of urbanization lies in the transformation of human settlements, characterized by rural-to-urban population migration accompanied by structural changes in industry and shifts in production and lifestyle patterns. Within the political economy framework, labor force represents human capital, and Smart Productivity—essentially an advanced form of productivity in the contemporary era—requires substantial human resources to facilitate its development. Urbanization supplies cities with significant rural migrant labor, thereby promoting the advancement of Smart Productivity. Additionally, the industrial structural transformation inherent in urbanization promotes technological advancement, contributing to the emergence of innovation-driven development models and the enhancement of Smart Productivity levels. The mediation mechanism is visualized in Fig 2.

Accordingly, **Hypothesis 2** is formulated: Increased Urban Density leads to higher levels of urbanization, which in turn increases the Smart Productivity.

### 2.3 Regional Heterogeneity

In China, provincial capitals and directly administered municipalities are typically regarded as the economic, cultural, and political centers of their respective regions. They hold a significant proportion of the provincial economic status and population size, and their urban development, planning models, and the urban issues they face are often highly representative.

The impact of urban density on Smart Productivity exhibits spatial heterogeneity across Chinese cities due to their distinct geographical characteristics and development trajectories. The eastern regions of China, characterized by larger populations and earlier implementation of economic liberalization policies, have emerged as the nation’s primary centers of economic development. In these regions, urban land use has become highly intensive to facilitate economic efficiency. However, continued emphasis on compact urban development may potentially diminish urban Smart Productivity efficiency due to negative externalities such as congestion effects. In summary, the provincial capitals and directly administered municipalities in the eastern region (such as Beijing and Shanghai) represent the most economically developed and densely urbanized cities in China. Their characteristics include a high-density urban development model, but they also face challenges such as environmental pollution and resource scarcity, which may have a negative impact on new-type productivity.

The central region of China is an area with significant economic development potential and strong growth momentum. For example, Wuhan, as the largest city in central China, serves as an important center for science, education, and transportation. Similarly, Zhengzhou and Changsha play vital economic and logistics roles in the region. The issue of urban compactness faced by cities in the central region is more complex than that of cities in the eastern region. Although the level of urbanization is gradually increasing, these cities opened up to the outside world later, are geographically deeper inland, and have relatively outdated planning concepts and lower levels of economic development. Many cities in central China still exhibit scattered spatial resource utilization, though transportation and infrastructure construction are gradually improving. The provincial capitals and directly administered municipalities in this region are in a phase of accelerated development. While their urbanization process has not yet reached the dense levels seen in the eastern region, they possess immense economic growth potential and substantial room for transformation in industries and green development. Therefore, urban density in the central region may have a positive impact on new-type productivity.

Compared to the eastern and central regions, urban development in the western region of China has generally been more delayed. However, in recent years, the Chinese government has vigorously supported the Western Development Strategy, leading to a gradual acceleration in the economic development of cities in the region. At the same time, most cities in the western region have lower urban density than those in the eastern region, which means that urban space utilization efficiency is relatively low. This is particularly evident in some medium-sized and small cities, as well as in remote areas, where urban expansion is more dispersed. There is still considerable room for improvement in infrastructure, public services, and other aspects.

Based on these spatial variations in urban development patterns and their potential implications, we propose Hypothesis 3: The relationship between urban density and Smart Productivity demonstrates significant regional heterogeneity.

**Fig 3:**
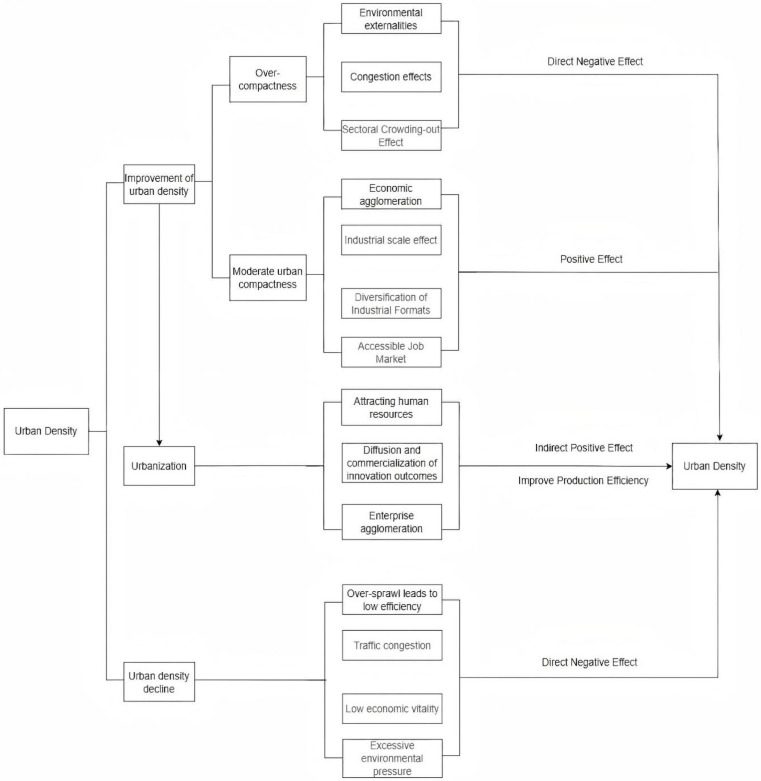
Theoretical framework.

The theoretical framework of this research is summarized in Fig 3.

## 3 Research design

### 3.1 Variable definitions

(1) Explained Variables

This study employs the level of new quality productivity as the explanatory variable. The conceptualization of new quality productivity encompasses several key transformations: the shift in production advantage criteria from purely “quantity-based” to “quantity and quality-based” factor inputs, the evolution from traditional production and manufacturing toward digitally interconnected integrated innovation, and the adherence to green development principles emphasizing energy conservation and consumption reduction [24]. Drawing from both the fundamental conceptualization of new quality productivity and relevant literature [24,32,33], this study constructs a comprehensive measurement framework using the entropy weight method. The framework evaluates new quality productivity through three primary dimensions: scientific and technological innovation productivity, green productivity, and digital productivity. For the Technological Innovation Productivity dimension, we included two secondary indicators: Innovation Capability and R&D Infrastructure. These were selected primarily because Technological Innovation Productivity should form a complete cycle encompassing inputs (funding, labor), processes (R&D activities), and outputs (patent numbers), thereby covering the entire process of technological innovation. Furthermore, based on the conceptual understanding that smart productivity should achieve key technological breakthroughs and innovations beyond traditional productivity [24], this study considers scientific and technological innovation as crucial indicators for measuring smart productivity, and has accordingly selected tertiary-level indicators.

Since existing concepts related to smart productivity also incorporate sustainability principles [24,27], Green Environmental Productivity was established as one of the primary indicators. We employed Industrial Emissions and Green and Environmental Protection as secondary indicators, aiming to achieve a balanced evaluation of green transformation outcomes through the combination of pressure indicators (emissions) and governance indicators (green spaces, environmental protection investments). These secondary indicators, Industrial Emissions and Green and Environmental Protection, were selected to align with our research objectives.

Existing research demonstrates that digital productivity constitutes another important dimension for measuring smart productivity [22]. The core of industrial digitalization lies in optimizing resource allocation, enhancing production efficiency, and accelerating technological innovation iteration through technological penetration and data-driven approaches. Achieving these objectives requires the support of digital infrastructure. Therefore, this study selected the level of digital infrastructure as one secondary indicator for measuring digital productivity. Simultaneously, assessing digital productivity also requires a comprehensive evaluation of the current state of urban digital industry development. Consequently, we chose the development status of urban digital industries (Digitalized Industry) as another secondary indicator for evaluating digital productivity. However, the construction of composite indices remains critically contingent upon researchers’ conceptual interpretations and operational definitions. The selection of dimensions and incorporation of specific indicators (e.g., blockchain technology applications) are frequently constrained by theoretical predispositions or data accessibility, potentially introducing selection biases. To mitigate such methodological limitations, this study employs measurement frameworks derived from established literature. Furthermore, to address potential distortions from single-indicator evaluations, we integrate three or more secondary indicators for each smart productivity dimension, thereby enhancing measurement robustness. The detailed indicator system is presented in Table 2.

**Table 2:** New-quality productive forces measurement.

First -level indicator	Second-level indicator	Third-level indicator	Computational method
Technological Innovation Productivity	Innovation Capability	Patent grants per Ten Thousand People	Patent grants per Capita
		R&D Input Intensity	R&D Expenditure/ Budgeted financial expenditure
	R&D Infrastructure	Total R&D Personnel	Total R&D Personnel in the Prefecture-Level City
		Internal R&D Expenditure	Internal R&D Expenditure in the Prefecture-Level City
		College Students per Ten Thousand People	Total College Students/ Capita in the Prefecture-Level City
Green Environmental Productivity	Industrial Emissions	Industrial Sulfur Dioxide Emissions	Sulfur Dioxide Emissions/ GDP
		Industrial Waste Gas Emissions	Industrial Waste Gas Emissions/ GDP
		Industrial Wastewater Emissions	Industrial Wastewater Emissions/ GDP
	Green and Environmental Protection	Green Coverage Rate	Green Coverage Area of Built-up Area/ Built-up Area
		Energy Conservation and Environmental Protection	Energy Conservation and Environmental Protection Expenditure/ GDP
Digital Productivity	Digital Infrastructure of Industry	Telecommunications Revenue per Capita	Telecommunications Revenue/ Urban Population
		Proportion of Employees in Computer Services and Software Industry	Number of employees in computer services and software industry/ Total number of employees
	Digitalized Industry	Internet Penetration Rate	Number of Internet Internet access/Urban Population
		Mobile Phone Subscribers Rate	The total number of mobile phone users/Urban Population

To ensure measurement objectivity, this study employs the entropy weight method to assign weights and quantify the new quality productivity measurement index system [16]. The reason for choosing the entropy weight method for measurement research in this study is that the entropy weight method is based entirely on the distributional characteristics of the data itself and does not rely on subjective judgement or pre-set model assumptions. By measuring the degree of dispersion (entropy value) of the information of each indicator, the entropy weight method can objectively reflect the amount of information provided by the indicator. In contrast, PCA and factor analysis may involve subjective selection of the number of factors when extracting the principal components or factors, introducing certain subjective factors; in addition, entropy weighting method can directly provide the weight of each indicator, reflecting its relative importance in the overall evaluation, which is easy to understand and make decisions, whereas the principal components or factors obtained by PCA and factor analysis tend to be a linear combination of multiple indicators, which, although it helps to achieve the dimensionality reduction analysis, but its economic or practical significance is not easy to understand intuitively. However, the weights of the entropy weighting method are completely determined by the degree of data dispersion, and if the distribution of variables is highly skewed or there are extreme values, it may lead to the weights deviating from the actual importance, for this reason, in this study, before carrying out the entropy weighting method of the data, it has been pre-empted according to the characteristics of the data to make the normalisation and inverse processing, in order to alleviate the problems that may be caused by this defect.

The methodological procedure encompasses the following sequential steps:

- Pre-processing of data for entropy weight method calculation.

First, the data are normalized and inverted:


$$Positive\;indicators:\;x=\frac{x_{ij}-\min_{j}}{\max_{j}-\min_{j}}+\lambda$$

$$Negative\;indicators:\;x=\frac{\max_{j}-x_{ij}}{\max_{j}-\min_{j}}+\lambda$$ (1)

In the formula, *x* is the data that were dimensionless using the polarization method, max_j_, min_j_are maximum and minimum value of *j* index.

- Calculation of information entropy using the entropy method.

$$e_{j}=-\frac{1}{\ln n}\sum_{i=1}^{n}\frac{x}{\sum_{i=1}^{n}x}\ln\left(\frac{x}{\sum_{i=1}^{n}x}\right)$$ (2)

*e*_*j*_ is the information entropy of *j* index; *i* denotes the sample city *i*; *x* is the indicator data after dimensionless processing.

- Calculation of the weights of the indicators.

$$\omega_{j}=\frac{1-e_{i}}{\sum_{i=1}^{m}1-e_{i}},j=1,2,3\ldots \ldots ,m$$ (3)

$\omega_{j}$ *ω*_*j*_ is the weight of index *j*, *i* is the sample city *i*,e_i_ is the information entropy of city *i*.

- Calculating Smart Productivity of cities.

$$\mu =\sum_{i=1}^{n}\omega_{j}*x$$ (4)

*ω*_*j*_ is the weight of index *j*, *i* is the sample city *i*, *μ* is Smart Productivity of a city, *x* is the indicator data after dimensionless processing.

(2) Core explanatory variables.

This study employs Urban Density as the primary explanatory variable. To ensure scientific rigor in data measurement, we construct a comprehensive indicator system based on established literature [14,36,37]. The Urban Density measurement framework encompasses four fundamental dimensions: economic compactness, population compactness, land use compactness, and transportation compactness. Similarly, to address the subjective errors in the selection of indicators associated with the calculation of the composite index, it was possible to reduce measurement errors by drawing on the literature and considering the multiple perspectives that should be included in the measurement of urban density. The detailed indicator system is presented in Table 3.

**Table 3:** Urban density measurement.

First-level Indicator	Second-level Indicator	Computational method
Economic Compactness	GDP Density	District GDP per Urban Area
	Fixed Asset Investment Ratio	District Fixed Asset Investment/District GDP
	Secondary and Tertiary Industry Value Added to GDP Ratio	Share of Secondary and Tertiary Industries in GDP
Population Compactness	Urban Population Density	Urban Population/Urban Area
	Non-agricultural Employment Ratio	Share of Secondary and Tertiary Industries in Total Employment
	Employment Density	Employment/ Urban Area
Land Use Compactness	Land Utilization Rate	Built-up Area/ Constructed Land Area
	Constructed Land per Capita	Constructed Land Area/ Urban Population
	Residential Land Ratio	Residential Land Area/ Constructed Land Area
Transportation Compactness	Buses per Ten Thousand People	Buses per Urban Population at Year-end
	Taxis per Ten Thousand People	Taxis per Urban Population at Year-end
	Road Area per Capita	Urban Road Area/ Capita

To ensure measurement objectivity, this study similarly employs the entropy weight method for weight assignment and quantification of the Urban Density index system.

(3) Mediating Variable

Based on the theoretical framework presented above, this study employs urbanization level as the mediating variable. To ensure scientific rigor and data accessibility, the urbanization level is measured using municipal district urbanization rates.

(4) Control Variables

Drawing from existing literature [24,38] and in accordance with this study’s research objectives, four control variables were selected.

A Government Fiscal Decentralization.

Under the current fiscal decentralization system, local governments possess greater autonomy in fiscal allocation. However, existing research presents divergent findings regarding the effectiveness of government fiscal decentralization. One stream of scholarship suggests that while fiscal decentralization promotes local economic growth in China, it simultaneously serves as an institutional driver of unsustainable economic development patterns [39], potentially impeding long-term regional productivity growth. Conversely, another body of research argues that the fiscal decentralization system enables local governments to enhance resource allocation efficiency, facilitate factor mobility, and promote technological innovation through the attraction of high-caliber human capital and investment [40], thus positively influencing Smart Productivity development. In this study, we measure the degree of government fiscal decentralization using the ratio of government budgeted revenues to expenditures.

B Industrial Structure Advancement.

The degree of industrial structure optimization reflects a region’s economic development foundation, whereby more sophisticated industrial categories and advanced production technologies influence Smart Productivity development. Industrial structure, measured by Composite Index=(Primary Industry*1)+(Secondary Industry*2)+(Tertiary Industry*3) the Industrial Structure Advancement, is hypothesized to have a positive correlation with Smart Productivity levels.

C Environmental Regulation.

Environmental regulation encompasses governmental policy measures aimed at preventing and controlling environmental pollution and enhancing environmental quality, as well as the constraining influence of social organizations and the public on polluters’ emission behaviors [41]. Environmental regulation increases the costs associated with short-term innovation failure, significantly influencing corporate decision-making and potentially inhibiting corporate green innovation initiatives. In this study, environmental regulation is operationalized through the comprehensive utilization rate of general industrial solid waste, which is hypothesized to have a negative relationship with Smart Productivity levels.

D Market Openness.

Higher levels of market openness promote local economic development through enhanced investment and talent acquisition. Additionally, the entry of technologically advanced foreign enterprises may catalyze a “learning effect” among local firms, stimulating technological innovation and advancement. This study employs the actual utilization of foreign capital as a measure of market openness, which is hypothesized to have a positive association with Smart Productivity levels.

### 3.2 Model construction and data sources

(1) Model construction.

According to the above analysis, the model is constructed as follows:


$$Model\;1:\;{NQPF}_{it}=\alpha_{0}+\alpha_{1}{COMP}_{it}+\alpha_{2}{TI}_{it}+\alpha_{3}{EN}_{it}+\alpha_{4}{OPEN}_{it}++\alpha_{4}{FAI}_{it}+\varepsilon_{it}$$ (5)

NQPF is Smart Productivity(Smart Productivity), COMP is Urban Density, TI is upgrading of industrial structure, EN is environmental regulation, OPEN is market openness, FAI is Government fiscal decentralization, ε is randomized perturbation term, α_0_-α_5_are parameter to be estimated, *i* and *t* represent city and time. This paper focuses on *t*he dynamic impacts over time rather than static differences between cities. The key explanatory variable, Urban Density, exhibits a significant temporal trend during the sample period. Employing a fixed-effects model would absorb its intertemporal variation, preventing the model from capturing the dynamic effects of this core variable. Therefore, we prioritize controlling for time-fixed effects to accurately identify the driving mechanisms of variables as they change over time. Additionally, considering the potential influence of prior Urban Density on current Smart Productivity, we conduct a robustness check by introducing a one-period lag of the Urban Density variable in the regression analysis to enhance the reliability of the results.


$$Model\;2:\;M_{it}=\beta_{0}+\beta_{1}COMP+\beta_{2}x_{it}+\varepsilon_{it}$$ (6)

$$Model\;3:\;{NQPF}_{it}=\gamma_{0}+\gamma_{1}{COMP}_{it}+\gamma_{2}M_{it}+\gamma_{3}x_{it}+\varepsilon_{it}$$ (7)

In formula 6, *M*_*it*_is the mediating variable, i.e., the level of urbanization. *x*_*it*_ represents four control variables. In this paper, we first test the mediating effect and conduct regression analysis according to model 2 to test whether there is a relationship between Urban Density and urbanization level. If *β*_*1*_, *γ*_*2*_ are significant,the mediation effect can be considered significant, if*γ*_*0*_is not significant, it can be considered as a full mediation effect, if *γ*_*0*_is significant, A partial mediation effect can be inferred when these conditions are met. In cases where stepwise regression results lack statistical significance, further analysis employing both Sobel and Bootstrap tests is conducted to assess the presence of mediating effects.


$$Model\;4:\;y=\beta_{0}+\beta_{1}x+\beta_{2}x^{2}+\varepsilon$$ (8)

In Equation 8, x represents the explanatory variable of urban compactness, *x*² denotes the quadratic term of urban compactness, *β₁* is the first-order coefficient, and *β₂* is the second-order coefficient. For the inverted U-shaped relationship to hold, the following conditions must be satisfied: the first-order coefficient *β₁* > 0 and the second-order coefficient *β₂* < 0.

(2) Research Scope and Data Description.

This study encompasses China’s provincial capital cities and municipalities directly under the central government. These urban centers are characterized by large populations, high industrial concentration, and superior scientific and innovative capabilities within their respective regions. Driven by national policy initiatives, these cities are positioned to become regional development hubs and serve as core engines of regional economic growth. However, Lhasa, Yinchuan, and Lanzhou were excluded from the analysis due to substantial data limitations.

The primary data sources include the China Urban Statistical Yearbook (2011–2022) and individual city statistical yearbooks, from which we obtained the following variables: District GDP, Secondary and Tertiary Industry Value Added to GDP Ratio, Urban Population, Total number of employees, Urban Area, Road Area per Capita, Buses per Ten Thousand People, Fixed asset investment, Patent grants, R&D Expenditure,Budgeted financial expenditure, Total R&D Personnel, Green Coverage Area of Built-up Area.

Data regarding Built-up Area, Constructed Land Area, and Residential Land Area were sourced from local natural resources bureaus and housing and construction bureaus. Environmental indicators, including Industrial sulfur dioxide emissions, Industrial Waste Gas Emissions, and Industrial Wastewater Emissions, were collected from local ecology and environment bureaus, supplemented by statistical yearbooks.

Energy Conservation and Environmental Protection data and College Students per Ten Thousand People were obtained from municipal government documents and local statistical yearbooks. The CNRDS China Research Data Platform and government documents provided data on: Green and Environmental Protection, Telecommunications Revenue, Proportion of Employees in Computer Services and Software Industry, Internet penetration rates, Mobile Phone Subscribers Rate.

For clarity, the data sources utilized in this study have been systematically cataloged in Table 4.

**Table 4:** Research data sources.

Variable Name	Data Sources
District GDP	China Urban Statistical Yearbook (2011–2022) and individual city statistical yearbooks
Secondary and Tertiary Industry Value Added to GDP Ratio	
Urban Population	
Total number of employees	
Urban Area	
Road Area per Capita	
Buses per Ten Thousand People	
Fixed asset investment	
Patent grants	
R&D Expenditure	
Budgeted financial expenditure	
Total R&D Personnel	
Green Coverage Area of Built-up Area	
Built-up Area	Local natural resources bureaus and housing and construction bureaus
Constructed Land Area	
Residential Land Area	
Industrial Sulfur Dioxide Emissions	Local ecology and environment bureaus
Industrial Waste Gas Emissions	
Industrial Wastewater Emissions	
Energy Conservation and Environmental Protection	Municipal government documents and local statistical yearbooks
College Students per Ten Thousand People	
Green and Environmental Protection	The CNRDS China Research Data Platform and government documents
Telecommunications Revenue	
Proportion of Employees in Computer Services and Software Industry	
Internet penetration rates	
Mobile Phone Subscribers Rate	
Market Openness	State Statistics Bureau
Environmental Regulation	China-Urban-Statistical-Yearbook (2011–2022)
Industrial Structure Advancement	
Government Fiscal Decentralization	

To ensure methodological rigor, market openness data underwent logarithmic transformation to minimize data distortion. Missing values were addressed through interpolation and ARIMA methodologies. Descriptive statistics for all variables are presented in Table 5.

**Table 5:** Descriptive statistics.

Variable Name	Sample Size	Minimum Value	Maximum Value	Mean	Standard Deviation	Median
Smart Productivity (New-Quality Productive forces)	308	0.095	0.524	0.266	0.077	0.257
Urban Density	308	0.107	0.654	0.301	0.104	0.291
Market Openness	308	6.795	16.835	14.061	1.683	14.515
Environmental Regulation	308	0.234	1.0	0.829	0.195	0.910
Industrial Structure Upgrading	308	2.278	2.836	2.527	0.116	2.523
Government Fiscal Decentralization	308	0.261	1.105	0.709	0.165	0.711
Urbanization in Municipal districts	308	0.440	0.976	0.738	0.111	0.737

## 4 Empirical results and analysis

**Fig 4:**
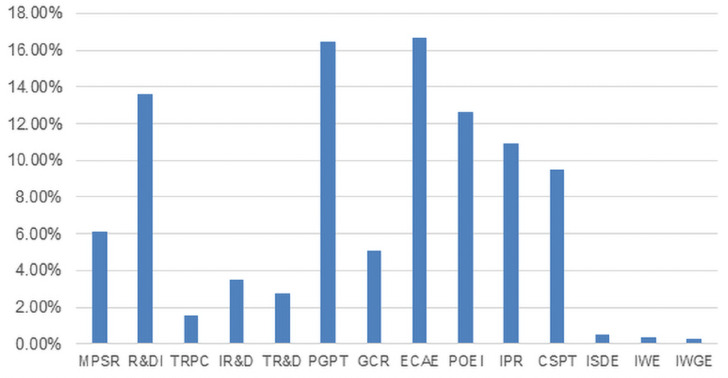
Calculation Results of Smart Productivity Weights. Note: MPSR ,Mobile Phone Subscribers Rate; R&DI,R&D Input Intensity; TRPC, Telecommunications Revenue per Capita; IR&D, Internal R&D Expenditure; TR&D, Total R&D Personnel ; PGPT, Patent grants per Ten Thousand People ;GCR, Green Coverage Rate ;ECAE, Energy Conservation and Environmental Protection; POEI, Proportion of Employees in Computer Services and Software Industry; IPR, Internet Penetration Rate; CSPT, College Students per Ten Thousand People; ISDE, Industrial Sulfur Dioxide Emissions ;IWE, Industrial Wastewater Emissions; IWGE, Industrial Waste Gas Emissions.

**Fig 5:**
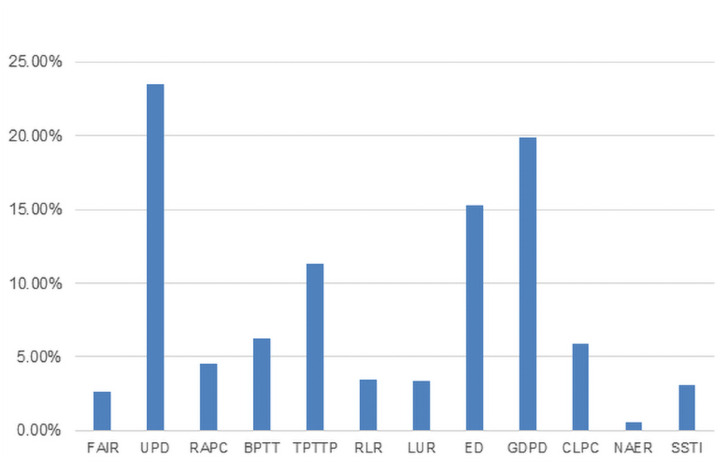
Urban Density Weight Calculation Results. Note: FAIR, Fixed Asset Investment Ratio; UPD, Urban Population Density; RAPC, Road Area per Capita; BPTT, Buses per Ten Thousand People; TPTTP, Taxis per Ten Thousand People; RLR, Residential Land Ratio; LUR, Land Utilization Rate; ED, Employment Density; GDPD, GDP Density; CLPC, Constructed Land per Capita; NAER, Non-agricultural Employment Ratio; SSTI, Share of Secondary and Tertiary Industries in GDP.

**Fig 6:**
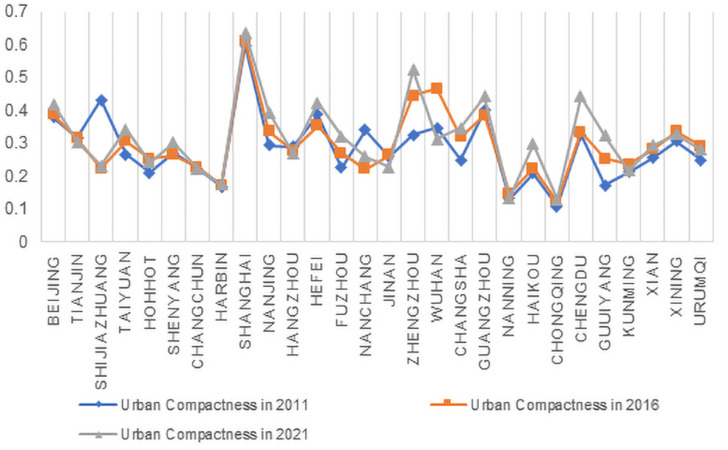
Urban Density Numerical Change.

**Fig 7:**
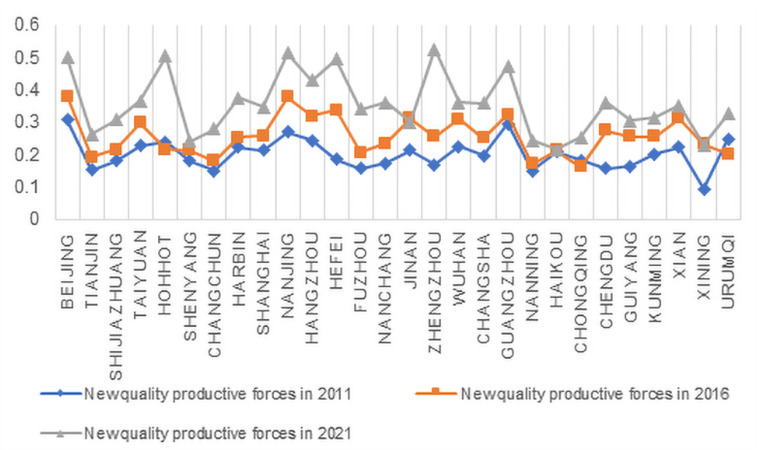
Smart Productivity Change.

Using the entropy weighting method, the weights of the Urban Density and Smart Productivity indicators were calculated (Fig 4 and Fig 5). Based on these weights, the specific scores for Urban Density and Smart Productivity were obtained. The scores for the years 2011, 2016, and 2021 were selected and visualized in Fig 6 and Fig 7. By comparing the two tables, it can be observed that, except for a few cities showing declining trends, the levels of Urban Density and Smart Productivity generally exhibited a synchronous upward trend over the 11-year period. This provides a solid data foundation for further analyzing the mechanism through which Urban Density influences Smart Productivity in the following section.

To comprehensively understand the relationship between the independent variables and the dependent variable in the dataset, and to mitigate issues arising from sample selection bias—thereby accurately capturing the relationship between the independent variables and the dependent variable—a full-sample regression is conducted using Equation (5) for each dataset.

First, a covariance test is performed on the explained variables, core explanatory variables, and control variables in Equation (5). The results show that the correlation coefficients are all below 0.8, indicating that there is no significant covariance among the variables, This validates the robustness of the empirical analysis of Hypothesis 1.

A panel data regression analysis was conducted using Equation (5). The F-test yielded a P-value of 0, and the Hausman test produced a P-value of 0.000 (Table 6). These results indicate the presence of endogeneity when employing the random effects model. To address this issue and avoid potential calculation errors, the fixed effects model was applied for the regression analysis. The regression results, presented in Table 7, reveal that Urban Density has a positive and statistically significant effect on the Smart Productivity of cities at the 1% significance level, with a coefficient of 0.11. This suggests that Urban Density promotes the level of Smart Productivity.

**Table 6:** Hausman test results.

	Fixed	Random	Difference	Std. err.
COMP	0.1143473	0.1041139	0.0102334	0.00251
EN	−0.0465592	−0.050342	0.0037827	0.0013729
TI	0.1660968	0.2902487	−0.1241519	0.0177663
FAI	0.0727331	0.0137186	0.0590145	0.0093008
OPEN	0.0082248	0.0108033	−0.0025784	0.0004132
Test of H0: Difference in coefficients not systematic				
Prob > chi2 = 0.0000				

**Table 7:** Regression results.

Variable Name	Results
COMP	0.1143473^***^(3.38)
EN	−0.0465592^***^(−2.97)
TI	0.1660968^***^(4.99)
OPEN	0.0082248^***^ (4.18)
FAI	0.0727331^***^ (3.28)
_cons	−0.3172387^***^(−3,61)
R^2^(within)	0.3661
F	33.73^***^
Sample Size(N)	308

Note: ***, **, * represent significance at the 1%, 5%, and 10% levels, respectively; the values in parentheses under the FE model regression results are t-values.

To account for potential lag effects that might influence the credibility of the regression results, the model was further extended by incorporating a one-period lag of the Urban Density variable. The regression outcomes, as shown in Table 8, indicate that the effect of Urban Density on Smart Productivity is not significant (P > 0.1) when considering a one-period lag. Given the enduring impact of spatial structural adjustments, the analysis was extended by incorporating a three-period lag of the original Urban Density data. This approach revealed a significant positive effect of Urban Density on Smart Productivity. These findings suggest that spatial structural adjustments, such as land-use mixing and transportation network optimization, may require a longer time frame (e.g., 3–5 years) to influence Smart Productivity through mechanisms like knowledge spillovers and industrial agglomeration. For instance, the formation of an innovation ecosystem necessitates prolonged interaction between enterprises and research institutions, which may not yield immediate effects in the short term (one-period lag).

**Table 8:** Lag effect analysis results.

Variable Name	Results
COMP	0.0727374(0.58)
COMP _lag1	0.0600349(0.48)
EN	−0.0472979^***^(−2.80)
TI	0.1544869^***^(4.26)
OPEN	0.0082556 ^***^ (3.98)
FAI	0.0731551^***^ (3.05)
_cons	−0.288434^***^(−3.01)
R^2^(within)	0.3610
F	24.86^***^
Sample Size(N)	280

Note: ***, **, * represent significance at the 1%, 5%, and 10% levels, respectively; the values in parentheses under the FE model regression results are t-values.

**Fig 8:**
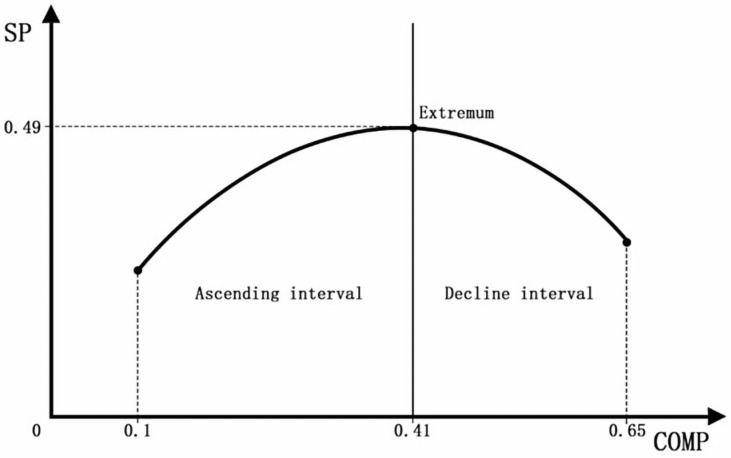
Inverted U-shaped relationship testing and visualization.

Building on these findings and the assumption of an inverted U-shaped relationship between urban density and smart productivity, as illustrated in Fig 1, we conducted formal validation of this nonlinear pattern. As shown in Table 9, the lower and upper bounds (0.106–0.654) define the critical range for the inflection point. The calculated inflection value of 0.4175972 for the COMP indicator falls within this interval, statistically confirming the inverted U-shaped relationship. Furthermore, the graphical representation in Fig 8 visualizes this relationship across urban density values of 0.1–0.65, revealing peak smart productivity (SP = 0.49) at the density threshold of 0.41.

**Table 9:** Inverse U-shaped Test Results.

	Lower bound	Upper bound
Interval	0.1069917	0.6540989
Slope	0.5675574	−0.4321504
Overall Test p-value	0.0000124^***^	
Overall Test t-value	4.29	
Extreme point	0.4175972	

Note: ***, **, * denote significance at the 1%, 5%, and 10% levels, respectively.

The comprehensive regression analysis (Table 9) demonstrates that surpassing this critical threshold (0.41) triggers negative impacts on smart productivity. Excessive urban density may induce systemic urban challenges, including traffic congestion, population overload, and environmental degradation. These compounding issues degrade urban livability and competitiveness, potentially leading to talent outflow, reduced foreign investment, and labor market imbalances characterized by shortages of both entry-level workers and high-skilled professionals.

To verify the robustness of the inverted U-shaped relationship, we performed bidirectional winsorization on urban compactness metrics and reconstructed quadratic term data. Subsequent utest analysis (Table 10) reaffirmed statistical significance (t = 4.04, p < 0.001), confirming the stability and reliability of the identified nonlinear pattern.

**Table 10:** Inverse U-shaped test results.

	Lower bound	Upper bound
Interval	0.1226184	0.6212633
Slope	0.5538189	−0.3899404
Overall Test p-value	0.0000348^***^	
Overall Test t-value	4.04	
Extreme point	0.4152343	

Note: ***, **, * denote significance at the 1%, 5%, and 10% levels, respectively.

The results, displayed in Table 9, indicate that the P-value of the inverted-U test is less than 0.01, confirming the existence of an inverted-U relationship between Urban Density and Smart Productivity, thereby supporting Hypothesis 1.

According to the regression results presented in Table 7, factors such as industrial structure upgrading, market openness, fiscal decentralization, and environmental regulation are all significant at the 1% level. Market openness has a positive effect on Smart Productivity, with a coefficient of 0.008. This suggests that the influx of foreign investment introduces advanced production technologies and management practices, leading to a “learning effect” that stimulates local technological and institutional innovation. Similarly, the positive regression coefficient of 0.16 for industrial structure upgrading indicates its role in enhancing Smart Productivity. Optimizing the industrial structure fosters economic growth, promotes the transition toward high-tech industries, and drives urban innovation.

The regression coefficient for environmental regulation is negative(−0.04), primarily because the implementation of local green policies tends to impose stricter constraints on high-polluting enterprises. Consequently, this reduces production and investment in green innovation, thereby hindering the development of Smart Productivity. In contrast, the regression coefficient for government fiscal decentralization is positive(0.07), indicating a favorable influence on Smart Productivity. This suggests that increased fiscal decentralization enables more flexible allocation of financial resources at the local level, which helps attract a skilled workforce and high-tech industrial investments. These factors foster knowledge spillovers and scale effects, ultimately enhancing regional scientific and technological innovation.8

### 4.2 Robustness test

(1) Alternative Measurements.

The regression results(Table 11) indicate that the Winsorized Urban Density variable exhibits a significant positive effect on Smart Productivity at the 1% level. Additionally, the control variables EN, TI, FAI, and OPEN are all significant at the 1% level, with their coefficient signs consistent with the original regression.

**Table 11:** Core explanatory variable data double-tailed trimming test.

Variable Name	Results
COMP_Winsor	0.119216^***^(3.48)
EN	−0.0004671^***^(−2.98)
TI	0.1652349^***^(4.97)
FAI	0.0723212^***^ (3.26)
OPEN	0.0081632^***^(4.15)
_cons	−0.3152291^***^(−3.6)
R2(within)	0.3675
F	33.93^***^
Sample(N)	308

Note: ***, **, * denote significance at the 1%, 5%, and 10% levels, respectively.

(2) Robust regression.

Given the potential influence of outliers on regression outcomes, a robust regression analysis was conducted, with the results presented in Table 12. The findings indicate that the variable Urban Density maintains a significant positive effect on Smart Productivity at the 1% level. Furthermore, the statistical significance and directional effects of the control variables—EN, TI, FAI, and OPEN—remain consistent with the original regression results, each exhibiting significance at the 1% level and retaining their respective coefficient directions. These outcomes further validate the robustness of the regression analysis.

**Table 12:** Robustness Test.

Variable Name	Results
COMP	0.1143473 ^***^(3.22)
EN	−0.04656^**^(−3.05)
TI	0.1660968^***^(6.43)
FAI	0.0727331^***^ (9.44)
OPEN	0.0082248 ^***^(10.00)
_cons	−0.3172387^***^(5.47)
R^2^(within)	0.3661
F	425.03^***^
Sample(N)	308

Note: ***, **, * denote significance at the 1%, 5%, and 10% levels, respectively.

### 4.3 Analysis of mediating effects

This study employs the bootstrap method to examine mediating effects, with the results presented in Table 13 and Table 14. Table 13 displays the mediating effect analysis results, revealing that the level of urbanization mediates the relationship between Urban Density and urban Smart Productivity. The first column reports the regression analysis of the explanatory variable COMP on the mediating variable URB, indicating a regression coefficient of 0.519 for COMP (p < 0.01), which demonstrates that COMP significantly and positively influences URB. The second column presents the analysis of the effects of both COMP and URB on the dependent variable SP, where COMP exhibits a regression coefficient of 0.143 (p < 0.01) and URB a coefficient of 0.265, both of which are significantly positive. These results suggest that URB serves as a mediator in the influence of COMP on SP.

**Table 13:** Mediation effect model test.

	URB	SP
Constant	0.581*** (34.072)	0.027 (1.052)
COMP	0.519*** (9.690)	0.143*** (3.448)
URB		0.265*** (6.835)
*R* ^2^	0.235	0.259
Fixed *R* ^2^	0.232	0.254
*F*	*F* (1,306)=93.896,*p* = 0.000	*F* (2,305)=53.210,*p* = 0.000

Note: ***, **, * denote significance at the 1%, 5%, and 10% levels, respectively.

**Table 14:** Bootstrap Test Result.

Item	c Total Effect	a	b	a*b Mediation Effect Value	a*b (Boot SE)	a*b (*z*)	a*b (*p*)	a*b (95% BootCI)	c’ Direct Effect
COMP=>URB=>SP	0.281***	0.519***	0.265***	0.138	0.031	4.438	0.000	0.131 ~ 0.252	0.143**

Note: ***, **, * denote significance at the 1%, 5%, and 10% levels, respectively.

Table 14 provides the bootstrap test outcomes, where the coefficient ‘a’ represents the effect of COMP on URB, ‘b’ denotes the coefficient of URB in the mediating effect test, and c’ represents the coefficient of COMP in the overall mediating effect test. The product ab reflects the indirect effect in the mediating analysis. The bootstrap results indicate that the overall effect is significantly positive, and the indirect effect (ab) has a p-value of 0.000 with a 95% confidence interval that does not include zero, thereby affirming the robustness of the mediating effect test.

Furthermore, higher urban density fosters the formation of industrial-scale effects, enhances economic vitality, diversifies industrial structures, and attracts an influx of rural labor into cities, thus accelerating the urbanization process. As an advanced form of productivity, smart productivity relies on a sufficient labor supply to drive its development.

Urbanization plays a pivotal role in this process by facilitating the migration of rural laborers to urban areas, ensuring an adequate labor supply for fostering Smart Productivity. Enhanced economic vitality attracts enterprises, thereby increasing demand for high-quality human capital and improving the urban talent pool. This influx of skilled labor leads to a concentration of talent, fostering knowledge spillovers that drive technological innovation and knowledge creation—key drivers of Smart Productivity.

Furthermore, this process creates a positive feedback loop: increased economic vitality attracts more labor, which in turn fosters scientific and technological advancements, further elevating Smart Productivity. Simultaneously, urbanization facilitates the optimization of industrial structures, enabling cities to transition from low-technology industries to more advanced, innovation-driven sectors. This transformation ultimately supports the development of an innovation-led growth model, strengthening Smart Productivity. Based on this, the validity of Hypothesis 2 can be confirmed.

### 4.4 Regional heterogeneity

The previous section conducted an overall regression on the impact of urban density on smart productivity, confirming that urban density has an inverted “U”-shaped effect on new productivity. However, The cities analyzed in this study are located across different regions of China, where geographical location, government policies, and other regional factors may influence the relationship between Urban Density and Smart Productivity. For instance, cities in eastern China benefit from a superior geographical location, greater openness to the outside world, and more advanced urban development concepts, positioning them at the forefront of urban planning and construction. In contrast, cities in central and western regions may exhibit different dynamics due to varying levels of development and regional characteristics.

To account for regional heterogeneity in the impact of Urban Density on Smart Productivity, the full dataset was divided into three subsamples: East, Central, and West. The classification aligns with China’s official regional standards and the geographic characteristics of provincial capitals and municipalities. Specifically, the eastern region includes 13 cities: Beijing, Tianjin, Shenyang, Changchun, Harbin, Shijiazhuang, Shanghai, Nanjing, Hangzhou, Fuzhou, Jinan, Guangzhou, and Haikou. The central region comprises six cities: Taiyuan, Hefei, Nanchang, Zhengzhou, Wuhan, and Changsha. The western region consists of Nanning, Chongqing, Chengdu, Guiyang, Kunming, Xi’an, Xining, Urumqi, and Hohhot. Fixed-effects regression was applied to each subsample using formula (5), and the results of the heterogeneity analysis are presented in Table 15.

**Table 15:** Regional Heterogeneity Regression Results.

Variable Name	Eastern Region	Central Region	Western Region
COMP	−0.1021101 *(−1.86)	0.156911 **(2.43)	0.1974641 ***(2.88)
CONTROL	control	control	control
_cons	−0.4866964 (−3.54)	−0.2538421(−1.10)	−0.3301065(−1.42)
R^2^(within)	0.4720	0.2550	0.3788
F	22.71***	3.42 ***	10.12 ***
N	143	66	99

Note: ***, **, * denote significance at the 1%, 5%, and 10% levels, respectively.

The findings of this study reveal significant regional disparities in the influence of Urban Density on Smart Productivity across China, alongside the associated positive and negative consequences. Specifically, Urban Density demonstrates a positive and statistically significant effect on Smart Productivity at the 5% level in the central and western regions. In contrast, in the eastern region, Urban Density has a negative impact on Smart Productivity, with a statistically significant effect at the 10% level. The impact coefficient for the western region (0.19) surpasses that of the central region (0.15), indicating that while Urban Density in the western region remains relatively low, the potential for future development is higher due to weaker agglomeration effects of industries, economic activities, and populations.

In the central region, the effect of urban density on smart productivity is significantly positive at the 5% level. Cities such as Changsha, Wuhan, and Zhengzhou exhibit high population densities, elevated economic activities, and robust industrial agglomeration effects. However, the large population sizes and advanced development levels in these cities appear to moderate the positive impact of urban density on smart productivity. Conversely, in the eastern region, the effect of urban density on smart productivity is significantly negative at the 10% level. Rapid urbanization following economic reforms has led to intense land development and diversified land use patterns. Nevertheless, local government-imposed restrictions on the expansion of construction land have produced a highly constrained urban land market, thereby limiting further population and enterprise agglomeration. Consequently, these constraints have hindered improvements in productivity and production efficiency in the region. Based on these findings, Hypothesis 3 is confirmed.

## 5 Discussion

### 5.1 Key findings

**Fig 9:**
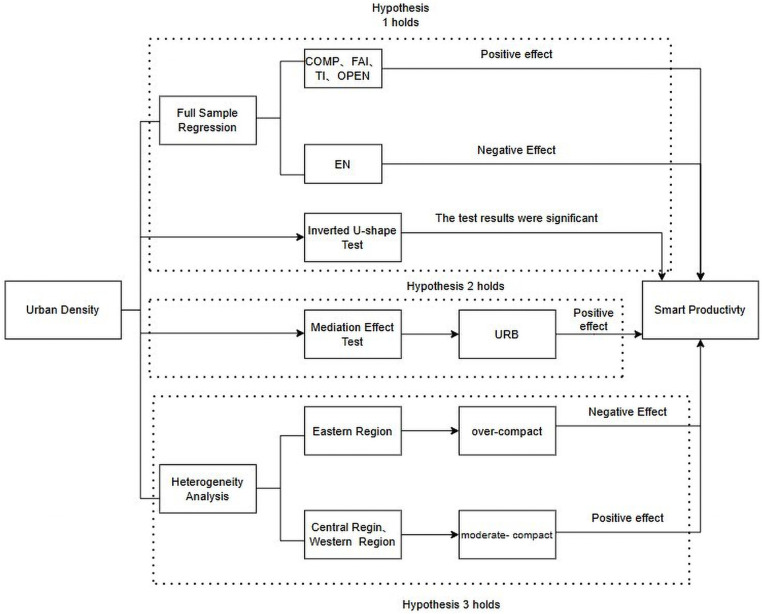
Summary of conclusions.

Based on panel data from China’s provincial capital cities and municipalities directly governed by the central government from 2011 to 2021, this paper investigates the mechanisms through which Urban Density influences Smart Productivity, leading to the following key findings. First, fixed-effects regression results for the full sample reveal an inverted “U”-shaped relationship between Urban Density and Smart Productivity, suggesting that the effect of density on productivity diminishes beyond a certain point. Second, mediation analysis demonstrates that urbanization level acts as a mediating factor between Urban Density and Smart Productivity. Specifically, higher urbanization levels positively contribute to Smart Productivity and facilitate the growth of new productive forces. Third, heterogeneity analysis indicates regional disparities in the relationship between Urban Density and Smart Productivity. In particular, Urban Density positively impacts new productive forces in China’s central and western regions, with a more pronounced effect in the western region. Conversely, in the eastern region, Urban Density exerts a negative influence on Smart Productivity. The conclusion is summarized in Fig 9.

### 5.2 Policy recommendations

Based on the findings of the empirical analysis, the following recommendations are proposed:

First, the development of compact cities tailored to local conditions should be promoted. Our analysis reveals that the relationship between urban density and smart productivity follows an inverted “U” shape. Consequently, in cities with low urban density and inefficient land use, efforts should concentrate on enhancing compact city construction to leverage the benefits of economic agglomeration and industrial scaling, ultimately boosting productivity and fostering economic growth. In new urban expansion areas, a design strategy characterized by “narrow roads and dense road networks” should be implemented, accompanied by the establishment of regional energy centers to improve the efficiency of public service facilities. Conversely, in cities with already high urban density, indiscriminately pursuing compact city construction may exacerbate problems such as overcrowding, traffic congestion, and ecological degradation. To mitigate these issues, urban management strategies must prioritize timely infrastructure upgrades, moderate adjustments to urban land expansion policies, controlled expansion of construction land to ease population pressure, real-time monitoring of high-pollution industries, and the safeguarding of urban ecological stability. Moreover, to accurately identify critical intervention nodes, the deployment of an urban efficiency monitoring system is recommended—for instance, by installing integrated sensor nodes or conducting periodic manual surveys at regular intervals to continuously collect metabolic data on energy, transportation, and waste. If the system’s efficiency declines by more than 5% over three to four consecutive months or falls below predetermined thresholds based on local conditions, an alert should be triggered to enable prompt measures to enhance urban operational efficiency or address potential ecological crises. Collectively, these measures can mitigate the adverse effects of excessive agglomeration, such as environmental deterioration and overcrowding, while effectively postponing the inverted “U” turning point through high-quality urban governance.

Second, promote the optimization of industrial structure and the development of emerging industries. The beneficial impact of industrial structure optimization on cities at various levels of economic development underscores the “new” dimension of Smart Productivity, driven by digitalization and intelligent systems. By deepening the role of labor inputs and enhancing the technological sophistication of production tools, such optimization elevates enterprise productivity. Local governments should actively foster the growth of emerging digital industries—such as artificial intelligence and big data—to cultivate an ecosystem of mass entrepreneurship and innovation.

To further stimulate widespread entrepreneurship and innovation while enhancing labor quality, it is essential to strengthen collaborations between high-tech enterprises and academic institutions. For instance, corporate CTOs could concurrently serve as university researchers, co-developing graduate programs tailored to technological needs and establishing dedicated scholarship initiatives. In highly developed urban clusters in the Yangtze River Delta, Pearl River Delta, and Beijing-Tianjin-Hebei regions, efforts should focus on creating high-technology hubs and scaling AI computing clusters to drive the digital transformation of traditional industries. Conversely, in central and western regions, governments could allocate special transition funds to reform traditional industry models, providing targeted subsidies for equipment investments and personnel training to support enterprises transitioning to digital industries.

Concurrently, enterprises are encouraged to optimize their internal organizational structures, increase investments in research and development, cultivate technical talent, and enhance overall innovation capabilities. Additionally, firms with technological advantages should be motivated to open access to their foundational patents, with each utilized patent eligible for government subsidies proportionate to its market value, thereby accelerating technological diffusion. For small and medium-sized enterprises, policies promoting collaborative research and development—such as shared laboratory facilities and joint access to research outputs—should be implemented. Achieving specific thresholds in equipment usage or research output could trigger government subsidies, thus easing the technological upgrade burden for these enterprises.

Third, optimize enterprise evaluation mechanisms and promote green innovation. Although current environmental regulations have a negative impact on Smart Productivity, the government should strive to balance sewage charges and environmental protection subsidies. This objective can be achieved by refining green credit eligibility criteria and enhancing the sewage charge mechanism to motivate enterprises toward green innovation. For example, a graded green credit access policy could be established: high-pollution industries would be eligible only for emergency loans with appropriately increased interest rates—and with a portion of the loan designated for green technology upgrades—whereas low-carbon industries might benefit from reduced interest rates and relaxed repayment terms. Additionally, the provision of targeted subsidies for green innovation, such as advance subsidies for critical green technology innovation, can mitigate associated risks, thereby encouraging enterprises to adopt environmentally sustainable practices and further stimulate innovation.

Fourth, advance urbanization to attract high-quality talent. Increased urban density plays a pivotal role in facilitating urbanization, which, in turn, contributes to the development of smart productivity. Policies should aim to lower barriers to rural labor migration while introducing preferential measures to attract high-caliber talent to urban areas. Enhancing urban living conditions by improving public service infrastructure, providing comprehensive social services, and safeguarding the rights of rural laborers is essential for drawing both labor and skilled professionals. For instance, in mega-cities, reducing the household registration points requirement for individuals who have accumulated a specified duration of employment in new towns situated at a certain distance from the city center could be considered; whereas in small and medium-sized cities, offering housing purchase or rental subsidies for 1–3 years during employment may be effective. Such measures are anticipated to facilitate talent agglomeration, stimulate knowledge spillover effects, and drive technological innovation within enterprises, ultimately advancing smart productivity.

Fifth, reform local government performance evaluation systems. Fiscal decentralization has been shown to negatively impact cities with lower levels of economic development. To address this, adjustments should be made to improve the coordination between central and local governments, ensuring more effective central oversight and regulation. Furthermore, local government performance evaluation systems must be restructured to align with the principles of high-quality development and the connotation of Smart Productivity. This includes incorporating evaluation indicators related to green innovation, environmental protection, and production efficiency. Such reforms can reduce “short-sighted” governance behaviors, fostering greater accountability and intrinsic motivation among local governments to enhance Smart Productivity.

### 5.3 Research limitations and future directions

#### 5.3.1 Current limitations.

This study employs the entropy weight method to measure urban density and intelligent productivity. While this approach enhances the objectivity of the results, the potential influence of extreme values in the dataset on computational outcomes requires further investigation. Subsequent research on urban density and intelligent productivity could integrate subjective weighting methods such as the Analytic Hierarchy Process (AHP) to strengthen the scientific validity of findings. Regarding potential subjectivity in indicator selection for measuring urban density and intelligent productivity, this research has mitigated such effects through comprehensive literature review and theoretical analysis of existing measurement frameworks.

The empirical analysis utilizes urban panel data from 2011 to 2021. Notably, data from 2020 to 2021 were collected during the global pandemic. Although preliminary analysis suggests minimal impact on key findings, the possibility of subtle influences cannot be entirely excluded.

The investigation reveals an inverted U-shaped impact mechanism of urban density on intelligent productivity in Chinese provincial capitals and municipalities. From an economic development perspective, these cities operate at mid-to-high international levels, suggesting the potential applicability of findings to cities with comparable economic status globally. Demographically, the studied cities predominantly feature populations exceeding 3 million, including several megacities with over 10 million residents, indicating probable relevance to international cities of similar scale. However, the applicability of conclusions to medium and small cities remains debatable due to sample limitations.

#### 5.3.2 Future research agenda.

In the methodology section, a combined approach that integrates subjective (human-assigned) weighting with objective weighting methods is adopted to ensure the objectivity of the computational results. With respect to the research scope, a holistic understanding of Urban Density and Smart Productivity development across China remains insufficient—particularly in small and medium-sized cities, which require further investigation. Moreover, spatial analysis tools, such as ArcGIS, will be employed to better incorporate urban spatial dimensions into the study. Techniques like Geographically Weighted Regression (GWR) and Geodetector will be applied to analyze the impact of Urban Density on Smart Productivity across cities of various scales throughout China.
